# The influence of scene context on object recognition is independent of attentional focus

**DOI:** 10.3389/fpsyg.2013.00552

**Published:** 2013-08-20

**Authors:** Jaap Munneke, Valentina Brentari, Marius V. Peelen

**Affiliations:** ^1^Center for Mind/Brain Sciences, University of TrentoTrento, Italy; ^2^Department of Cognitive Psychology, Vrije UniversiteitAmsterdam, Netherlands

**Keywords:** object recognition, natural scene processing, high-level vision, semantic consistency, spatial attention

## Abstract

Humans can quickly and accurately recognize objects within briefly presented natural scenes. Previous work has provided evidence that scene context contributes to this process, demonstrating improved naming of objects that were presented in semantically consistent scenes (e.g., a sandcastle on a beach) relative to semantically inconsistent scenes (e.g., a sandcastle on a football field). The current study was aimed at investigating which processes underlie the scene consistency effect. Specifically, we tested: (1) whether the effect is due to increased visual feature and/or shape overlap for consistent relative to inconsistent scene-object pairs; and (2) whether the effect is mediated by attention to the background scene. Experiment 1 replicated the scene consistency effect of a previous report (Davenport and Potter, 2004). Using a new, carefully controlled stimulus set, Experiment 2 showed that the scene consistency effect could not be explained by low-level feature or shape overlap between scenes and target objects. Experiments 3a and 3b investigated whether focused attention modulates the scene consistency effect. By using a location cueing manipulation, participants were correctly informed about the location of the target object on a proportion of trials, allowing focused attention to be deployed toward the target object. Importantly, the effect of scene consistency on target object recognition was independent of spatial attention, and was observed both when attention was focused on the target object and when attention was focused on the background scene. These results indicate that a semantically consistent scene context benefits object recognition independently of the focus of attention. We suggest that the scene consistency effect is primarily driven by global scene properties, or “scene gist”, that can be processed with minimal attentional resources.

## INTRODUCTION

The human visual system is extraordinarily adept at detecting, categorizing, and naming objects embedded in natural scenes. The properties of this ability have been studied extensively (Henderson and Hollingworth, 1999; Bar, 2004; Torralba et al., 2006; Fabre-Thorpe, 2011; Wolfe et al., 2011). Many objects are usually found in specific contexts: a car is found on a road, a deer in a forest. Prior research has shown that the availability of scene context (i.e., a semantically consistent background) facilitates the detection and recognition of objects within that scene (Biederman et al., 1982; De Graef et al., 1990; Joubert et al., 2007; but see Hollingworth and Henderson, 1998). However, the precise mechanisms responsible for this facilitative effect remain elusive. A better understanding of these mechanisms is crucial for gaining further insight into how objects and scenes are interactively processed by the visual system.

Convincing evidence for the influence of scene context on object processing was provided by studies that manipulated the semantic consistency of target objects and the natural scenes they were presented in. Such studies present target objects in either semantically consistent (e.g., a microwave in a kitchen) or semantically inconsistent (e.g., a microwave in a forest) natural scenes. Effects of semantic consistency between scene and object stimuli have been found using eye movement measurements (Loftus and Mackworth, 1978; Henderson et al., 1999; Brockmole and Henderson, 2008; Vo and Henderson, 2009, 2011), behavioral measurements (Davenport and Potter, 2004; Joubert et al., 2007; Fize et al., 2011), and electrophysiological measures (Ganis and Kutas, 2003; Mudrik et al., 2010). Furthermore, effects of scene context on object processing have been reported for multiple levels of object processing, ranging from the rapid detection of superordinate object categories (e.g., animals; Fize et al., 2011) to the naming of objects at the subordinate level (Davenport and Potter, 2004). These tasks differ in many ways. For example, animal detection likely involves the matching of incoming visual information to an attentional “template” of animal-diagnostic shape features to inform a quick present/absent decision (Duncan and Humphreys, 1989; Treisman, 2006). By contrast, in object naming tasks, the to-be-named object is not known before stimulus onset, and successful task performance relies on detailed recognition of the object (e.g., recognizing a person as a priest; Davenport and Potter, 2004). Given these differences, it is plausible that scene context affects these tasks in different ways. In the present study, we focus on the effect of scene context on the naming of objects.

Important evidence for the facilitative effect of scene context on object naming comes from a study by Davenport and Potter (2004). In their study, participants were presented with natural scenes comprised of a background scene with a foreground object pasted into it. The object could be semantically related to the scene background (e.g., a priest in a church) or could lack this semantic relationship (e.g., a priest on a football field). The scene containing the object was presented for a brief duration (80 ms), ensuring that any effects observed were not due to eye movements. At the end of each trial, participants were asked to type in the name of the perceived foreground object, with the background scene being irrelevant to the task. The results of the study showed that participants responded more accurately to an object when presented in a semantically consistent scene compared to a semantically inconsistent scene, showing that, despite being task irrelevant, the background was processed to an extent such that it influenced processing of the target object. The first aim of the current study was to replicate the findings of Davenport and Potter (2004) to establish the reliability of these findings, and to test whether they generalize to another language and population (Italian).

**FIGURE 1:**
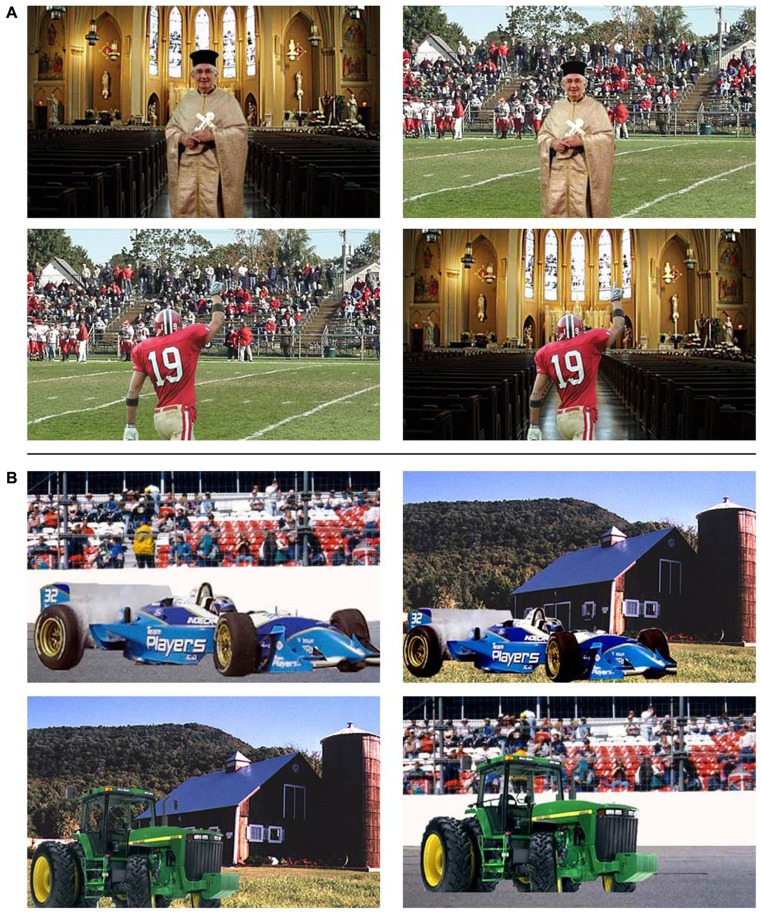
**Example stimuli from the original Davenport and Potter (2004) study and Experiment 1.** Each object is presented in a consistent (left column) and inconsistent (right column) setting. **(A)** Example stimuli showing more overlap in low-level visual features between target object and background scene in the consistent scenes. For example, when presented on a semantically consistent background the color of the priest’s robes matches the color of the church wall and its ornaments. Similarly, the red-white clothing of the American football player matches the color of the crowd and other players in the background. When presented in an inconsistent setting such overlap is reduced. **(B)** Example stimuli containing large background objects (e.g., a farm) that may have been attended prior to identifying the target object, due to uncertainty about the location of the target object (e.g., whether it appeared to the left or right of fixation).

Despite the convincing results of Davenport and Potter (2004; which were successfully replicated in the current study), it is possible that the effect observed was not due to semantic influences of scene context, but rather to differential overlap of low-level visual features (such as color) and/or object shape between the background scene and the foreground object. For example, when a sandcastle (object) is presented onto a beach background (scene), object and background share a number of low-level features such as color and texture. In contrast, a scene consisting of a sandcastle presented on a field of grass does not contain this overlap in low-level features (see **Figure 1A** for additional examples). Note that this concern does not apply to the same degree to earlier studies addressing related questions using line drawings (e.g., Biederman et al., 1982). The goal of the current Experiment 2 was to rule out influences of differential visual and shape overlap and thus to provide a more stringent test of whether the *semantic* consistency of background scene and foreground object influences object naming.

A final aim of the current study was to investigate the influence of attentional focus on the scene consistency effect. In the study by Davenport and Potter (2004), and in the current Experiment 1, target objects were presented close to the center of the screen. However, their precise location was not known before stimulus onset. Therefore, participants would have initially attended the background scene while locating the target object. In Experiment 3, we tested whether attentive processing of the background scene is required for the scene consistency effect to emerge. Testing the influence of attention on the scene consistency effect could provide information about the types of scene properties that drive the scene consistency effect. For example, the processing of global scene statistics has been shown to be independent of attentional resources, unlike the identification of more detailed scene properties such as other objects in the scene (Ariely, 2001; Chong and Treisman, 2003).

## EXPERIMENT 1

The aim of this experiment was to replicate the consistency effect reported by Davenport and Potter (2004), using their original stimuli. As we employed the same experimental set-up and design, we expected to find that participants would recognize objects with a higher accuracy when presented on a semantically consistent background, compared to when the same object was presented on a semantically inconsistent background.

### MATERIALS AND METHODS

#### Participants

Twelve participants took part in this experiment. Participants’ age ranged from 24 to 35 years old (mean ± SD**= 27.8 ± 3.19 years; one male). All participants had normal or corrected-to-normal vision. Written informed consent was obtained prior to the start of the experiment. Participants were rewarded with course credit or a monetary reward.

#### Stimuli

Experiment 1 utilized the original stimulus set used by Davenport and Potter (2004) along with the same experimental design. Participants were presented with 28 images of natural scenes containing an object pasted into the foreground, in such a way that both the object and the background were clearly visible. On half the trials the natural scene contained an object that was semantically consistent with its background whereas the other half of the trials showed an object inconsistent with its background (see **Figures 1A,B**). Consistency of the scene-object pairing was counterbalanced over participants in such a way that half the participants would see a certain target object in a semantically consistent setting, whereas the other half of the participants would see the same object in a semantically inconsistent setting. All scenes were presented in the center of the screen and subtended a visual angle of 17.64° by 10.54°. Size and location of the target objects varied over the different natural scenes [average horizontal × vertical dimensions: 7.93° (SD = 3.64) × 7.35° (SD = 1.84)], but the foreground object was always clearly distinguishable as the target object.

#### Procedure

**FIGURE 2:**
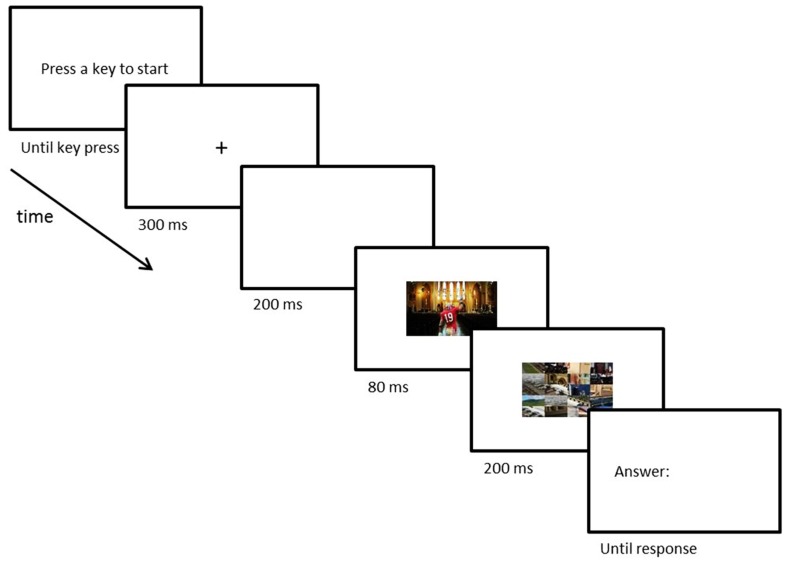
**Time course of a trial in Experiment 1.** After an initial fixation period, participants saw a briefly flashed and subsequently masked scene with an object pasted in the foreground. At the end of each trial, participants typed in the name of the presented foreground object.

Participants were seated in a dimly lit room at approximately 60 cm from the computer monitor. All stimuli were presented on a 19″ CRT monitor. **Figure 2** shows the time course of a typical trial.

The participants started each new trial manually by pressing any key. Once the trial started, participants were presented with a fixation-cross (300 ms) followed by a blank period (200 ms). After the blank period, the natural scene containing the object appeared (80 ms) immediately followed by a mask (200 ms). Masks consisted of a 4-by-5 grid of pieces of a random set of cut-up scenes that were never used as stimuli. Following the mask, the Italian word for “answer” (risposta) would appear and participants typed in the name of the target object. Participants were instructed to be as specific as possible when naming the object (e.g., “priest” rather than “person”). Responses were unspeeded and only checked for accuracy. Prior to the experiment participants performed six practice trials, using scenes and objects that were not used in the main experiment. Different from the original Davenport and Potter (2004) study, the current study was performed in the Italian language (as opposed to English).

### RESULTS AND DISCUSSION

**FIGURE 3:**
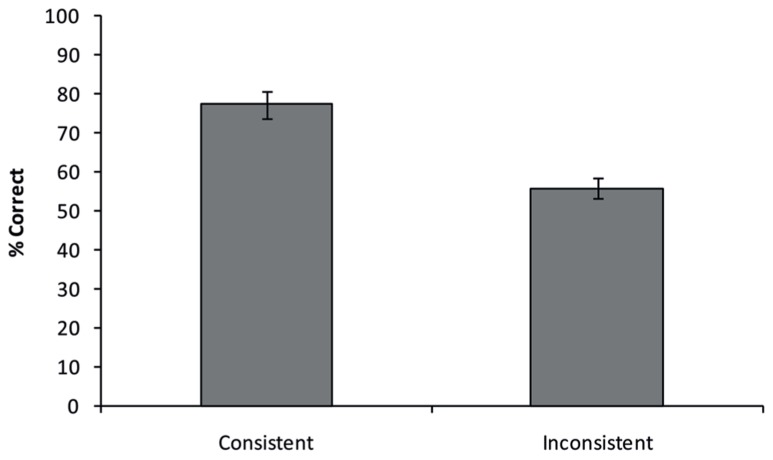
**Semantic consistency effect obtained in Experiment 1.** Participants were more accurate in recognizing objects presented on a semantically consistent background, compared to a semantically inconsistent background. Error bars reflect the standard error of the mean.

All answers provided by the participants were checked for accuracy by five independent raters who only saw the presented object, but not the background, ensuring no influence of semantic background in their ratings. Raters were instructed that only specific names should be considered correct (e.g., for the example in **Figure 1A**, “priest”, “pastor”, or “clergyman” would all be correct, whereas “person” would be incorrect). If three or more raters concluded that a given answer was correct, the answer was deemed as being correct. **Figure 3** shows the average percentage of correct responses for both consistent and inconsistent trials. A paired-samples *t*-test shows that participants responded more accurately on consistent then on inconsistent trials [77.4% vs. 56.0%; *t*(11) = 4.377, *p* = 0.001].

The results of Experiment 1 replicated the findings observed by Davenport and Potter (2004) and show that they generalize to another population and to naming objects in another language than English (Italian). When participants attempted to recognize an object in a briefly presented scene, the semantic consistency of the background affected perception of the sought-after object in such a way that a semantically consistent background led to higher accuracy in recognizing the target objects, even though the background was not directly relevant to the task.

## EXPERIMENT 2

**FIGURE 4:**
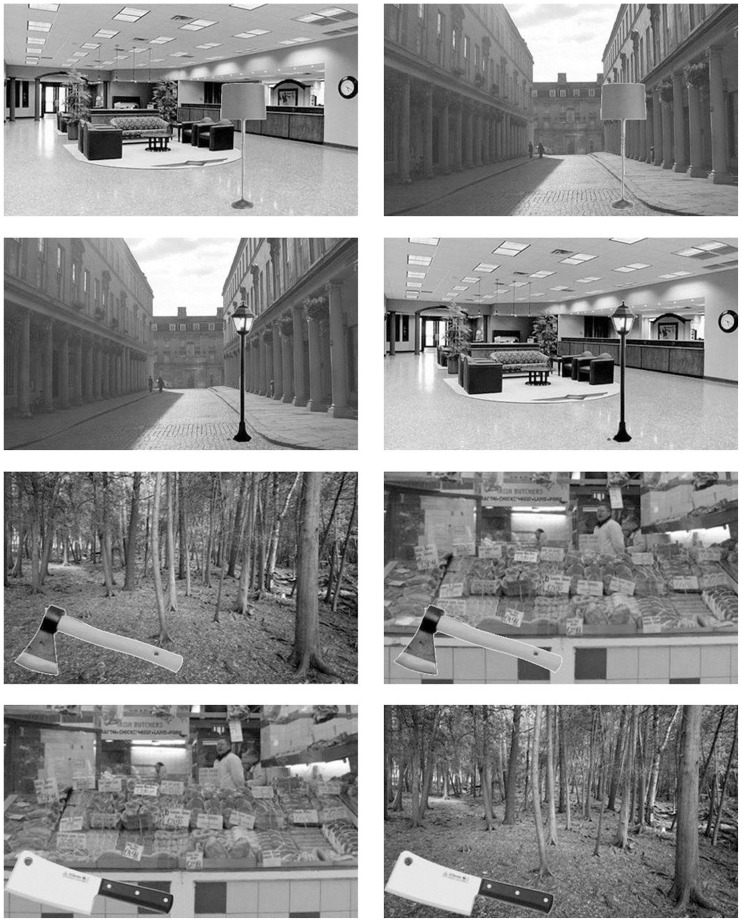
**Example stimuli used in Experiments 2, 3a, and 3b**.

Experiment 1 showed that scene context influences object processing. It is not clear from these results, however, which parts or properties of the background scene are responsible for the observed consistency effect. As outlined in the introduction, one possible reason for the consistency effect in Experiment 1 could be the greater overlap in shape and low-level features, such as color, between the consistent scene and the target object, as illustrated in **Figure 1A**. Experiment 2 was designed to investigate this possibility. To do so, a new stimulus set was created, again consisting of backgrounds with target objects pasted in the foreground. In order to control for the influence of overlap in color, all stimuli were converted to gray-scale. Additionally, objects and scenes were chosen in such a way that semantically consistent and inconsistent objects in a given scene shared an overall similar shape (see **Figure 4**). Finally, in order to enhance the influence of scene gist on object recognition, the inconsistent condition always consisted of an indoor scene paired with an outdoor object or an outdoor scene paired with an indoor object.

### MATERIALS AND METHODS

#### Participants

Fourteen participants (mean age ± SD**= 22.3 ± 3.41 years; three males) took part in the experiment. All participants had normal or corrected-to-normal vision. Written informed consent was obtained prior to the start of the experiment. Participants were rewarded with course credit or a monetary reward.

#### Stimuli

The design of Experiment 2 was highly similar to that of Experiment 1, with a number of crucial changes in the used stimuli. Participants were presented with 56 natural scenes (twice as many as in Experiment 1) containing a foreground object pasted onto a background. The foreground object was not necessarily placed or scaled to naturally fit in the background scene (**Figure 4**). Half the trials contained an object that was semantically consistent with its background whereas the other half would show an object inconsistent with its background (see **Figure 4**). Stimuli were controlled in a number of ways: to eliminate effects related to color, all images (objects, backgrounds, and masks) were converted to gray-scale images. Furthermore, the 56 target objects consisted of pairs of objects that shared a semantic relation and had roughly similar shapes (e.g., indoor and outdoor lamps; **Figure 4**). The average size of the target objects was somewhat smaller than the average size of the target objects used in Experiment 1 [average horizontal × vertical dimensions: 6.50° (SD = 3.30) × 6.19° (SD = 2.06)]. Finally, in order to maximize the effects of gist on object processing, backgrounds were chosen in such a way that on inconsistent trials, an outdoor background was always paired with an object normally found in an indoor setting or vice versa. For example, a street scene could be paired with either a streetlight (consistent) or a living room lamp (inconsistent), and these same objects were also presented in a living room setting. Half of the participants were shown the two objects in a semantically inconsistent context (e.g., a streetlight and living room lamp shown in living room and street, respectively) whereas the other half of the participants was shown the two stimuli in the consistent context.

#### Procedure

The procedure of Experiment 2 was identical to that of Experiment 1. Prior to the experiment participants performed eight practice trials, using scenes and objects not used in the main experiment.

### RESULTS AND DISCUSSION

**FIGURE 5:**
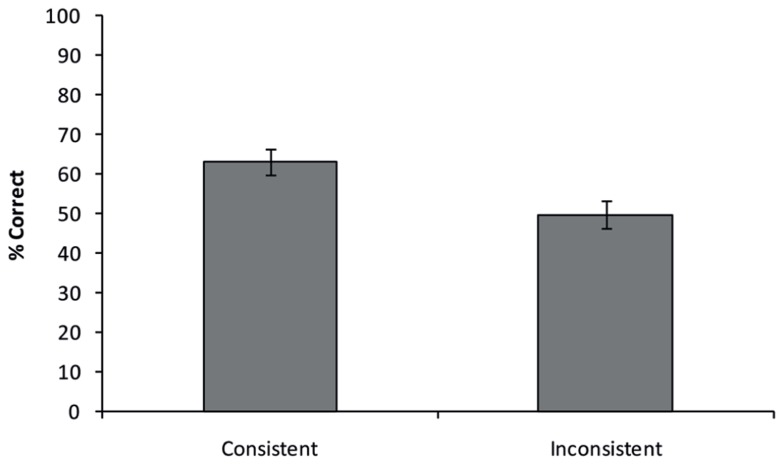
**Consistency effect obtained in Experiment 2.** Similar to Experiment 1, participants were more accurate in discriminating objects presented on a consistent background, compared to an inconsistent background. Error bars reflect the standard error of the mean.

Similar to Experiment 1, five independent raters scored all answers, using the same procedure and cut-off as in Experiment 1. **Figure 5** shows the average percentage of correct responses for semantically consistent and inconsistent trials.

A paired-samples *t*-test showed that participants were more accurate in naming an object presented on a consisted background compared to an object presented on an inconsistent background (Consistent: mean = 63.0%, Inconsistent: mean = 49.7%, *t*(13) = 4.053, *p* = 0.001). Thus, a strong consistency effect could still be observed with stimuli that were matched across conditions on visual feature and shape overlap between object and scene. This indicates that the observed consistency effect was most likely caused by semantic properties of the scene.

Despite finding a reliable consistency effect in both Experiments 1 and 2, the effect was numerically larger in Experiment 1 than in Experiment 2. This could be due to a number of differences between the two experiments, including differences in the low-level visual feature overlap (e.g., color) between scene and object, the matching of target shapes across consistent and inconsistent scenes in Experiment 2 but not Experiment 1, the size of the target objects, and particularly the specific background scenes and target objects that were used as stimuli – it is likely that the recognition of some objects (e.g., ambiguous objects) benefits more from scene context than the recognition of other objects, and that some scenes provide a stronger context than others. Future work is necessary to separately investigate the contribution of these factors.

A question that remains is whether attentive processing of the background scene is required for the semantic consistency effect. That is, does scene context influence object processing irrespective of attentional focus or does it influence object processing only when attention is (perhaps briefly) focused on the background scene, due to ambiguity concerning the location of the target object. Providing participants with knowledge concerning the location of the target object would allow focused attention to be deployed to the target location, drawing attention away from the background scene. Would reduced attention to the background scene reduce the semantic consistency effect? Experiments 3a and 3b addressed this question by employing two different cueing procedures, providing the participant with knowledge about the target object’s location.

## EXPERIMENT 3a

The aim of Experiment 3a was to investigate the role of spatial attention in the semantic consistency effects observed in Experiments 1 and 2. Specifically, when the location of the target object is known in advance, would scene context still influence object processing?

### MATERIALS AND METHODS

#### Participants

Eighteen participants (mean ± SD**= 23.5 ± 3.85 years; three males) who did not take part in Experiment 2 took part in Experiment 3a. All participants had normal or corrected-to-normal vision. Written informed consent was obtained prior to the start of the experiment. Participants were rewarded by course credit or a monetary reimbursement.

#### Stimuli

The setup of Experiment 3a was highly similar to Experiment 2. Four additional stimuli were added to the stimulus set used in Experiment 2, resulting in a total of 60 stimuli. One major change to the design consisted of adding a location cue prior to the onset of the natural scene. The location pre-cue consisted of a small black cross (0.46° × 0.46°), presented on a white background. Participants were instructed to covertly attend the location cue, while remaining fixated on the center of the screen. Location cues were indicative of the location (estimated center of gravity) of the target object on 2 out of 3 trials (~67% valid). The invalid cues were presented at the same vertical position as the valid cue, but mirrored over the vertical meridian of the scene, resulting in a horizontally shifted cue toward the opposite visual hemifield. Due to the target objects being relatively large foreground objects pasted into the scene, invalid cues generally did not lead to ambiguity as to which object functioned as the target object. For those scenes in which this was nonetheless the case, the cue was horizontally shifted until it no longer fell on a possible alternative target object. Semantic consistency and cue validity were counterbalanced across participants, in such a way that each target object was presented in a semantically consistent and inconsistent setting as well as being cued validly or invalidly equally often.

#### Procedure

The procedure of this experiment was similar to the procedure used in the previous experiments. After the initial fixation, the location cue would be presented for 500 ms. The duration of the blank screen prior to the onset of the natural scene was extended from 200 to 500 ms in order to give the participants additional time to process the cue. Other than these changes trials were identical to Experiment 2.

### RESULTS AND DISCUSSION

**FIGURE 6:**
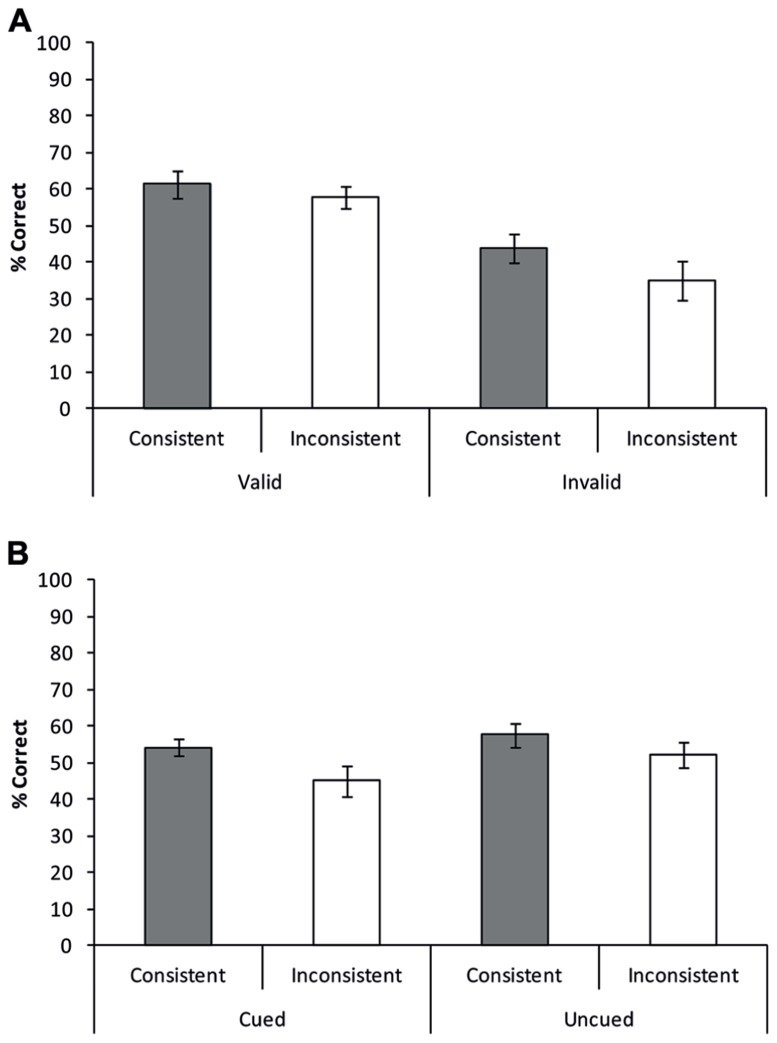
**Scene consistency effects as a function of attentional cueing to the target location in (A) Experiment 3a and (B) Experiment 3b.** Similar to Experiments 1 and 2, participants were more accurate in recognizing objects presented on a consistent background, compared to an inconsistent background. Importantly, scene consistency effects were equally strong for trials in which the location of the target was cued as for trials in which the location of the target object was not (Experiment 3b) or invalidly (Experiment 3a) cued. Error bars reflect the standard error of the mean.

Similar to Experiments 1 and 2, five independent raters, employing the same method and cut-off as in the previous experiments, rated all answers. A repeated measures ANOVA was performed with semantic consistency (consistent vs. inconsistent) and cue validity (valid vs. invalid) as factors (**Figure 6A**). Results showed a main effect of consistency with better overall performance for consistent compared to inconsistent trials [consistent: mean = 52.6% correct, inconsistent: mean 46.4% correct; *F*(1,17) = 7.123, *p* = 0.016]. A main effect of validity was also observed, showing a significantly higher accuracy when the location of a target object was validly cued compared to when it was invalidly cued (valid: mean = 59.6%, invalid: mean = 39.4%; *F*(1,17) = 9.275, *p* = 0.007). Importantly, no interaction between consistency and validity was observed [*F*(1,17) = 1.062, *p* = 0.317], indicating that the consistency effect was equally strong for validly and invalidly cued target objects. In other words, the effect of scene context on object recognition was not significantly attenuated when spatial attention was focused on the target object, relative to when attention was focused on part of the background scene.

## EXPERIMENT 3b

To extend and replicate the findings of Experiment 3a, a different cueing procedure was used in Experiment 3b. Instead of using attentional pre-cues, target location information in Experiment 3b was provided, on half the trials, by a salient red rectangle centered on the target object. Because all images were in grayscale, the red rectangle should pop out and capture attention (e.g., Theeuwes, 1992), immediately directing attention to the target object when the image was presented.

### MATERIALS AND METHODS

#### Participants

Fourteen participants (mean ± SD**= 23.2 ± 4.35 years; one male) who did not take part in Experiments 2 and 3a took part in Experiment 3b. All participants had normal or corrected-to-normal vision. Written informed consent was obtained prior to the start of the experiment. Participants were rewarded by course credit or a monetary reward.

#### Stimuli

The same stimulus set used in Experiment 3a was used in Experiment 3b. In order to guide attention toward the target object’s location, a salient red rectangle was presented around the target object providing participants with information regarding the target’s location. The simultaneous cue was presented on 50% of the trials. On the remainder of the trials no cue was presented. Cued items were semi-randomly chosen in such a way that half the trials that contained a cue consisted of consistent object-scene pairs, whereas the other half consisted of inconsistent object-scene pairs.

#### Procedure

The experimental procedure was identical to Experiment 2.

### RESULTS AND DISCUSSION

Similar to the previous experiments, five independent raters scored the answers given by the 14 participants. A repeated measures ANOVA with consistency (consistent, inconsistent) and cueing (cued, uncued) showed a main effect of consistency indicating that participants were more accurate in naming the target object when presented on a semantically consistent background, compared to when presented on a semantically inconsistent background [consistent: mean = 56.0% correct, inconsistent: mean = 48.8% correct, *F*(1,14) = 7.759, *p* = 0.015]. The main effect of cueing was not significant [*F*(1,13) = 3.583, *p* = 0.081]. Importantly, no interaction between consistency and cueing was observed (*F *< 1). In line with Experiment 3a the lack of an interaction shows that knowledge about the correct target location does not attenuate the consistency effect (**Figure 6B**).

## COMBINED ANALYSIS OF EXPERIMENTS 3a AND 3b

Experiments 3a and 3b suggest that target location ambiguity does not influence the size of the consistency effect. To strengthen the point that knowledge concerning the location of the target object does not attenuate (or facilitate) the semantic consistency effect, data from Experiments 3a and 3b were collapsed to increase statistical power (*N *= 32). Consequently an ANOVA was performed with two within-subjects variables: consistency (consistent, inconsistent) and cueing (cued, uncued). The uncued condition contains the invalid cue condition of Experiment 3a and the no-cue condition of Experiment 3b (in both cases, the target location was not cued). Additionally, cue-type (pre-cue, simultaneous cue), as defined by the two different experiments, was included as a between-subjects variable. Results of this analysis showed a main effect of Consistency [*F*(1,30) = 14.740, *p* = 0.001], with higher accuracy for consistent object-scene pairs, compared to inconsistent object-scene pairs (Consistent: mean = 54.3% correct, Inconsistent: mean = 47.6% correct). In addition, participants tended to respond more accurately when correct target location information was present compared to when this information was absent [*F*(1,30) = 3.550, *p* = 0.069; Present: mean = 54.7% correct, absent: 47.2% correct]. Correct target location availability interacted with the type of cue used [*F*(1,30) = 10.298, *p* = 0.003], showing facilitation when using a pre-cue, but a numerically opposite effect when using the simultaneous cue (**Figure 6**). No other interactions were observed, critically showing that knowledge about the target’s location did not influence the consistency effect (*F *< 1). Planned paired-samples *t*-tests showed that a consistency effect was observed both when target location was known [*t*(31) = 2.239, *p* = 0.032] and when target location was not known [*t*(31) = 2.617, *p* = 0.014].

The results of Experiments 3a and 3b converge to show that the scene consistency effect is independent of attentional focus; similar consistency effects were observed when the target object location was known as when it was not known. Thus, the background scene appears to influence object processing even when attention was not directed at the scene. This finding suggests that semantic consistency effects in the current experiments were primarily driven by scene properties that are, to some extent, independent of the focus of attention.

The main effect of validity in Experiment 3a shows that participants actively used the pre-cue to focus attention on the target object, thus verifying the effectiveness of the cueing manipulation. There may be several reasons why the simultaneous cue in Experiment 3b did not have the same facilitative effect. One possibility is that the benefit of the cue was offset by a masking effect of the red rectangle, with the salient red rectangle benefiting performance by drawing attention to the object’s location (and away from the scene) but also making the object somewhat harder to perceive. Alternatively, or additionally, attention may have been captured by the red rectangle itself and then redirected to the target object within the rectangle. In this scenario, attention in the cued condition would first be directed at the red rectangle before being directed at the target object, whereas in the uncued condition attention would initially be directed at the scene before being directed to the target object. Although in this case there would be no net benefit of the cue, the cue would nonetheless have directed attention away from the scene. Crucially, the magnitude of the consistency effect was similar regardless of the presence or the absence of a cue. Therefore, these results suggest that the consistency effect does not depend on attentive processing of the background scene.

## GENERAL DISCUSSION

The current study tested which aspects of the scene background drive the semantic consistency effect on object naming, as observed previously (Davenport and Potter, 2004) and in the current Experiment 1. A highly controlled stimulus set was used in order to gain further insight into the role of low-level visual and shape properties on the consistency effect (Experiment 2). In addition, the influence of focused attention was studied (Experiments 3a and 3b). Experiment 1 clearly replicated the effects observed by Davenport and Potter (2004), showing that participants were more accurate in naming a briefly presented object when it was placed on a semantically consistent background, as compared to a semantically inconsistent background. Compared to Experiment 1, more controlled stimulus set was used in Experiment 2, minimizing the effects of differential low-level visual feature and shape overlap between target object and background scene. Regardless of these changes, a strong consistency effect was also observed in Experiment 2. Therefore, it seems most likely that the observed scene-object interaction is based on the extraction of semantic information derived from the scenes.

Experiments 3a and 3b showed that even when participants had (prior) knowledge concerning the location of the presented object, a semantic consistency effect was still observed. No difference in the magnitude of the consistency effect was found when the location of the sought-after object was known, allowing focused attention to the target, compared to when its location was unknown, resulting in attending the background scene. These results indicate that the location of spatial attention does not influence the effects of scene context on object processing. Therefore, the semantic consistency effect appears not to require attentive processing of the background scene. This conclusion is in line with the results of Davenport and Potter (2004), who tested the semantic consistency effect both in an experiment in which only the foreground object had to be reported (the background scene could be ignored) and in an experiment in which both the background and the foreground object had to be reported. These experiments revealed similar semantic consistency effects, suggesting that actively reporting (and thus attending) the background scene was not required for it to affect the recognition of the foreground object. Furthermore, foreground objects were reported equally accurately in the experiment in which the background scene had to be reported as in the experiment in which the background scene was irrelevant, supporting the proposal that objects and scenes are processed interactively rather than in isolation (Davenport and Potter, 2004).

Which properties of the background scene might drive the semantic consistency effect? One way to frame this question is with respect to the distinction between local versus global scene properties. Global scene properties, such as a statistical summary of spatial layout properties, are thought to be processed rapidly and in parallel to local object processing (Oliva and Torralba, 2006). Importantly, the processing of global scene statistics requires minimal attentional resources (Ariely, 2001; Chong and Treisman, 2003), by contrast to local object processing. The current finding that semantic consistency effects occur independently of whether attention is located on the target object or on the background scene is consistent with the hypothesis that the semantic information is derived from a coarse global representation of the scene, such as its overall structure and coarse spatial layout. The processing of global scene properties may lead to what is often referred to as the “gist” of a scene (Potter, 1975; Greene and Oliva, 2009). Scene gist might be sufficient to activate a *scene schema* (Biederman et al., 1982; Schyns and Oliva, 1994), resulting in memory-guided predictions of objects commonly present in the activated scene schema, thereby facilitating object detection and recognition (Bar, 2004; Bar et al., 2006). Future experiments could further test the hypothesis that global scene properties drive the scene consistency effect, for example by manipulating the spatial frequency content of the scenes (global properties should be preserved in low-pass filtered scenes).

To summarize, object recognition is facilitated when the object is presented in a semantically consistent context. This effect cannot be attributed to overlap in low-level visual features or object shape between target object and scene background. The finding that contextual effects were observed for scenes and objects that are presented for merely 80 ms suggests that scene context is processed rapidly. Additionally the processing of scene context was largely independent of top-down spatial attention. Together, these results are consistent with the proposal that rapidly derived scene gist facilitates the recognition of objects that are semantically related to the scene (Bar, 2004; Torralba et al., 2006).

## Conflict of Interest Statement

The authors declare that the research was conducted in the absence of any commercial or financial relationships that could be construed as a potential conflict of interest
